# G-protein-coupled estrogen receptor activation upregulates interleukin-1 receptor antagonist in the hippocampus after global cerebral ischemia: implications for neuronal self-defense

**DOI:** 10.1186/s12974-020-1715-x

**Published:** 2020-02-01

**Authors:** Ning Bai, Quanguang Zhang, Wenli Zhang, Bin Liu, Fang Yang, Darrell Brann, Ruimin Wang

**Affiliations:** 1 Neurobiology Institute of Medical Research Center, North China University of Science and Technology, Tangshan, 063210 Hebei China; 2 Department of Neuroscience and Regenerative Medicine, Medical College of Georgia, Augusta University, Augusta, GA 30912 USA; 3 First Department of Neurology, Hospital Affiliated to North China University of Science and Technology, Tangshan, 063000 Hebei China; 4 Key Laboratory of Dementia and Cognitive Disorder in Tangshan, North China University of Science and Technology, International Science & Technology Cooperation Base of Geriatric Medicine of China, 21 Bohai Road, Caofeidian Xincheng, Tangshan, 063210 Hebei China

**Keywords:** Global cerebral ischemia, G-protein-coupled estrogen receptor 1 (GPER/GPR30), Inflammasome, NACHT-, LRR- and PYD-containing protein 3 (NLRP3), Interleukin-1 receptor antagonist (IL1RA)

## Abstract

**Background:**

G-protein-coupled estrogen receptor (GPER/GPR30) is a novel membrane-associated estrogen receptor that can induce rapid kinase signaling in various cells. Activation of GPER can prevent hippocampal neuronal cell death following transient global cerebral ischemia (GCI), although the mechanisms remain unclear. In the current study, we sought to address whether GPER activation exerts potent anti-inflammatory effects in the rat hippocampus after GCI as a potential mechanism to limit neuronal cell death.

**Methods:**

GCI was induced by four-vessel occlusion in ovariectomized female SD rats. Specific agonist G1 or antagonist G36 of GPER was administrated using minipump, and antisense oligonucleotide (AS) of interleukin-1β receptor antagonist (IL1RA) was administrated using brain infusion kit. Protein expression of IL1RA, NF-κB-P65, phosphorylation of CREB (p-CREB), Bcl2, cleaved caspase 3, and microglial markers Iba1, CD11b, as well as inflammasome components NLRP3, ASC, cleaved caspase 1, and Cle-IL1β in the hippocampal CA1 region were investigated by immunofluorescent staining and Western blot analysis. The Duolink II in situ *proximity ligation assay* (PLA) was performed to detect the interaction between NLRP3 and ASC. Immunofluorescent staining for NeuN and TUNEL analysis were used to analyze neuronal survival and apoptosis, respectively. We performed Barnes maze and Novel object tests to compare the cognitive function of the rats.

**Results:**

The results showed that G1 attenuated GCI-induced elevation of Iba1 and CD11b in the hippocampal CA1 region at 14 days of reperfusion, and this effect was blocked by G36. G1 treatment also markedly decreased expression of the NLRP3-ASC-caspase 1 inflammasome and IL1β activation, as well as downstream NF-κB signaling, the effects reversed by G36 administration. Intriguingly, G1 caused a robust elevation in neurons of a well-known endogenous anti-inflammatory factor IL1RA, which was reversed by G36 treatment. G1 also enhanced p-CREB level in the hippocampus, a transcription factor known to enhance expression of IL1RA. Finally, in vivo IL1RA-AS abolished the anti-inflammatory, neuroprotective, and anti-apoptotic effects of G1 after GCI and reversed the cognitive-enhancing effects of G1 at 14 days after GCI.

**Conclusions:**

Taken together, the current results suggest that GPER preserves cognitive function following GCI in part by exerting anti-inflammatory effects and enhancing the defense mechanism of neurons by upregulating IL1RA.

## Background

Global cerebral ischemia (GCI) is well known to result in significant neurological and cognitive defects in animals and humans [1]. GCI has multiple causes, including cardiac arrest, asphyxiation, and hypotensive shock. The hippocampal CA1 region is highly vulnerable to damage from GCI, resulting in significant delayed death of hippocampal pyramidal neurons [2, 3]. Currently, there are no effective therapies for preserving cognitive function after global cerebral ischemia. Therapeutic hypothermia has been used clinically, but a meta-analysis failed to find a strong benefit on survival or neurological outcome [4]. Thus, there is a clear need to find new potential therapies for preserving cognitive function after GCI.

In previous studies, we and others showed that activation of G-protein-coupled estrogen receptor (GPER) is strongly neuroprotective in an animal model of GCI [5–7]. GPER, also known as GPR30, is the most recently identified member of the estrogen receptor family. Beginning with studies published in 2000, it was found that GPER binds the potent estrogen, 17β-estradiol (E2), and helps mediate E2-induced rapid activation of extracellular regulated kinases (ERKs) and cAMP generation [8–10]. GPER is highly localized in the cerebral cortex and hippocampus of the brain, with strong expression also noted in the basal forebrain, thalamus, and dorsal striatum [5, 11]. Studies to explore the role and functions of GPER in the brain have employed G1, a specific agonist, and either G15 or G36, GPER antagonists [12, 13]. GPER shRNA and antisense oligonucleotide knockdown have also been employed in several studies [5, 14]. Intriguingly, we and others previously reported that GPER activation via G1 administration can rapidly activate PI3K-Akt and MEK-ERK rapid kinase signaling pathways in the hippocampus and exert strong neuroprotection against GCI [5–7]. We also showed that E2 neuroprotection against GCI could be abolished by GPER knockdown in the hippocampal CA1 region [5].

Neuroinflammation can also contribute significantly to neuronal cell death in neurodegenerative disorders and in cardiac arrest, and attenuation of neuroinflammation can be neuroprotective [15, 16]. An important advance in the neuroinflammation field was the identification of inflammasomes as critical proteins that trigger neuroinflammation [17]. NLRP3 inflammasome is the most studied inflammasome in the CNS. It is a multiprotein complex that mediates activation of caspase-1 and promotes secretion of pro-inflammatory cytokines, such as interleukin-1β (IL-1β) and interleukin-18 (IL-18) [18]. After activation and release, the biological actions of IL-1β are known to be mediated by the type 1 IL-1 receptor (IL-1R). Intriguingly, an endogenous IL-1 receptor antagonist (IL1RA) has been identified in neurons that can block IL-1β pro-inflammatory actions by competing with IL-1β for IL-1R binding. Thus, IL1RA has been reported to exert potent anti-inflammatory and neuroprotective actions in the brain and other tissues [19–21].

Since neuroinflammation is implicated to play a significant role in neurodegeneration, we hypothesized that GPER neuroprotective effects in GCI could be due, in part, to anti-inflammatory actions of GPER. We thus examined the ability of GPER activation to regulate microglial activation, NLRP3 inflammasome activation, and IL-1β production in the hippocampus after GCI. In addition, we examined whether GPER activation could regulate IL1RA in the hippocampus after GCI and determined whether IL1RA mediates the anti-inflammatory, neuroprotective, anti-apoptotic, and cognitive-enhancing effects of GPER following GCI.

## Materials and methods

### Antibodies and reagents

The following antibodies were used: NeuN (Millipore Biotechnology, MAB377); GPER (sc-48525); Iba1 (sc-32725), GAPDH (sc-32233), NLRP3 (Life science, Lot L27693), NLRP3 (ab4207), ASC (sc-22514-R), CD 11b (Gentex, GTX76060), cleaved caspase 1 (cell signaling, #4199S), cleaved IL1β (sc-7884), IL1RA (ab124962), and IL1RA blocking peptide (ab200257); and NF-κB-p65 (ab32568), H2A (ab177308), Bcl2 (sc-492), cleaved caspase 3 (D175, 5A1E), CREB (cell signaling, #9197X), and p-CREB (cell signaling, #9198S), tubulin (sc-9104). Alexa-conjugated secondary antibodies were from Molecular Probes/Invitrogen (Carlsbad, CA). Polyvinylidene difluoride (PVDF) membranes with pore size of 0.45 μm were from Millipore (USA). BCIP (5-bromo-4-chloro-3-indolyl-phosphate) and NBT (nitro blue tetrazolium) were from Promega (Madison, WI). TUNEL Kits (LOT #1639496, Life technologies) were from Active Motif Company. Duolink PLA kits (DUO92105), Duolink PLA Rabbit MINUS (DUO92003), and PLA Goat PLUS (DUO92005) proximity probes were from Sigma-Aldrich. Unless indicated otherwise, all the other chemicals were from Sigma-Aldrich (St. Louis, MO).

### Animal and GCI model

Adult female Sprague-Dawley rats (Beijing HFK Bioscience Co., Ltd., 3 months old) were housed in a temperature-controlled (22–24 °C) room with water and food freely available. All procedures used in this study were approved by the Institutional Animal Care and Use Committee of North China University of Science and Technology (Ref. 2016047), and were conducted in accordance with the guidelines of the National Natural Science Foundation of China for animal research. The rats were bilateral ovariectomized (OVX) under isoflurane anesthesia and then randomly allocated to each group. In order to reduce bias in the study, a double-blind procedure was carried out in which drug-treatment animals were administrated by blinding investigators and statistical analysis was blindly performed by the authors. A total of 170 rats were used through the study. Of the total number of rats that underwent global cerebral ischemia (GCI), 10 rats died in ischemia reperfusion, and 14 rats were eliminated from further experiment due to not meeting the established criteria for successful cerebral ischemia. GCI animal model was induced at 1 week after OVX by the four-vessel occlusion (4-VO) as our previous description [22, 23]. Briefly, the rats were anesthetized using chloral hydrate (350 mg/kg, i.p.), then both vertebral arteries were electrocauterized through the alar foramen of the first cervical vertebra and both common carotid arteries (CAA) were exposed followed by closing the incision with a suture. After 24 h, the animals were lightly anesthetized to re-expose and occlude the CAA for 12 min with aneurysm clips. Rats that lost their righting reflex within 30 s and whose pupils were dilated with unresponsive to light during ischemia were deemed as successful and selected for the experiments. Resumption of carotid artery blood flow was verified visually by releasing the clips. Rectal temperature was maintained at about 36.5–37.5 °C during the procedure with an incubator. For sham-operated animals, all rats were performed exactly as for ischemic animals except that the CCA were not clamped.

### Administration of drugs

GPER agonist, G1 (Tocris, Cat. No. 3577, 10 μg/day), or/and GPER antagonist, G36 (Tocris, Cat. No. 4759, 10 μg/day) was administrated subcutaneously using minipump (0.25 μl/h, Alzet Model 2004) beginning at the time of OVX surgery. For vehicle-treatment group, the same volume cottonseed oil with 1% DMSO was administrated at the same time-points as G1 or G36 administration. IL1RA antisense oligodeoxynucleotide (AS, 5′-ACCAGCTCATTGCTGGGTAC-3′) or scrambled missense (MS, 5′-CCGCGAAAATCGCTTTAGCA-3′) was synthesized by Integrated DNA Technologies, Inc. The last 3 bases on both the 5′ and 3′ were end-phosphorothioated to limit ODN degradation. IL1RA-AS or MS (10 nmol/day) was dissolved in 0.9% saline and was continuously injected into the left lateral ventricle (anteroposterior, 0.8 mm; lateral, 1.5 mm; depth, 3.5 mm; from bregma) beginning at 3 days prior to ischemia until the end of the experiments (Alzet minipump 0.5 μl/h, model 2002, or 1 μl/h, model 2001) using brain infusion kit (Alzet, Lot no 10331-14).

### Histological analysis

The rats were anesthetized using isoflurane at reperfusion 1 day, 3 days, and 14 days and transcardially perfused with 0.9% saline followed by 4% paraformaldehyde in 0.1 M phosphate buffer (PB, pH 7.4). The brains were removed and postfixed in the same fixative overnight at 4 °C, followed by dehydration in 30% sucrose in 0.1 M PB till completely subsidence of the tissues and then cut longitudinally into 25 μm sections with a cryostat. Coronal sections were collected through the entire dorsal hippocampus (2.5–4.5 mm posterior from bregma) to investigate the neuroprotective effect of GPER following GCI by performing in situ apoptosis detection using TUNEL kit as described by the manufacturer with minor modifications, simultaneously carrying out immunofluorescence staining for NeuN. Briefly, the sections were washed using PBS for 30 min, permeabilized with 0.4% Triton X-100-PBS for 1 h, blocked in 10% donkey serum for 1 h, and then incubated in anti-NeuN antibody (1:800) overnight at 4 °C. After rinsing three times over 30 min with 0.1% Triton X-100-PBS, the sections were incubated with secondary antibodies (Alexa Fluor 488-nm donkey anti-mouse IgG) at room temperature for 1 h followed by washing for 5 × 10 min in 0.1% Triton X-100-PBS. The following steps were protected from light. The sections were incubated in TdT reaction buffer A for 10 min and then in TdT reaction mixture including enzyme solution for 1 h at 37 °C. After 5 min of washing with dH_2_O, the sections were incubated in Click-iT Plus TUNEL reaction cocktail for 30 min at 37 °C, washed with 0.1% Triton 100-PBS over 20 min, and then mounted on slides covered with water-based mounting medium. Images were captured under laser scanning confocal microscopy (LSCM, Olympus FV1000), and analysis was carried out using Digital imaging software (FV10-ASW 1.5). The number of NeuN- or TUNEL-positive CA1 neurons per 250-μm length of the medial CA1 pyramidal cell layer was bilaterally counted in five or six sections of different animals. Cell counts from the right and left hippocampus on each of the seven or eight sections were averaged to provide the mean value. A mean ± SE was calculated from the data in each group and statistical analysis performed as described below.

### Immunofluorescence staining and confocal microscopy

The coronal sections (25 μm) at time-points of reperfusion at 3 days and 14 days were prepared. All steps including washing, permeabilizing, and blocking were the same as described in histological analysis. The sections were incubated in the following primary antibodies overnight at 4 °C: anti-GPER (1:100), anti-NeuN (1:800), anti-Iba1 (1:1000), anti-CD11b (1:1000), anti-cleaved-IL1β, anti-NLRP3 (1:100), anti-ASC (1:100), anti-IL1RA (1:200), p-CREB (1:200), or anti-NF-κB-p65 (1:200). After washing for 3 × 10 min with 0.1% Triton X-100-PBS, the sections were incubated with secondary antibodies (Alexa Fluor 568-nm donkey anti-mouse IgG, Alexa Fluor 568-nm donkey anti-rabbit IgG, Alexa Fluor 488-nm donkey anti-goat IgG, and Alexa Fluor 488-nm donkey anti-mouse IgG) at room temperature for 1 h, followed by a final washing for 5 × 10 min in 0.1% Triton X-100-PBS. If necessary, the nucleus was counterstained using mounting medium with DAPI (Lot ZA0210, Vector Laboratories, Inc. Burlingame, CA 94010). The confocal images were captured on a laser scanning confocal microscope (LSCM, Olympus FV1000) and digital imaging software (FV10-ASW 1.5 Viewer).

### Duolink II proximity ligation assay

The Duolink II in situ *proximity ligation assay* (PLA) immunoassay was performed as described previously by our group [22, 24]. Briefly, after the same processes of washing, permeabilizing, and blocking as histological analysis, cerebral coronal sections were incubated using anti-NLRP3 (1:100) and anti-ASC (1:100) primary antibodies overnight at 4 °C. The slides were then incubated with Duolink PLA Rabbit MINUS and PLA Goat PLUS proximity probes for 1 h at 37 °C. Ligation and amplification were carried out using the Duolink in situ detection reagent kit according to the manufacturer’s protocol. DAPI was used to counter stain the nucleus. Images were captured in the hippocampal CA1 region under FV1000 LSCM, and red spots represented the interactions between NLRP3 with ASC.

### Brain homogenates and subcellular fractionations

The rats were sacrificed under deep anesthesia at 3 days and 14 days after ischemia. The brains were quickly removed, and the hippocampal CA1 regions of the two sides were micro-dissected on an ice pad. The total cytosolic or nuclear protein fraction isolation was performed as described by our group previously [22]. In brief, the tissues were homogenized in 1-ml ice-cold homogenization buffer consisting of (in mM) 50 HEPES, pH 7.4, 150 NaCl, 12 β-glycerophosphate, 3 dithiotheitol (DTT), 2 sodium orthovanadate (Na_3_VO_4_), 1 EGTA, 1 NaF, 1 phenylmethylsulfonyl fluoride (PMSF), 1% Triton X-100, and inhibitors of proteases and enzymes (Thermo Scientific, Rockford, IL150825, USA) with a Teflon-glass homogenizer. The homogenates were centrifuged at 15,000*g* for 30 min at 4 °C to get a total fraction in the supernatants. When necessary, cytosol and nuclear fractions were extracted. Briefly, tissues were homogenized in ice-cold buffer A containing (in mM) 10 HEPES, pH 7.9, 1 DTT, 1 Na_3_VO_4_, and inhibitors of proteases and enzymes, and mixed and then allowed to swell on ice for 10 min. The tubes were vigorously vibrated for 30 s and centrifuged at 15,000*g* for 30 min at 4 °C. The supernatants contained the cytoplasm fraction, and the pellets were washed three times with buffer A and re-suspended in buffer B [(in mM) 20 HEPES, pH 7.9, 400 NaCl, 20% glycerine, 1 DTT, 1 Na_3_VO_4_] with inhibitors of proteases and enzymes. After adding NP-40 to 0.6% of total solution, the tubes were vigorously rocked at 4 °C for 30 min on a rotator and centrifuged at 12,000*g* for 15 min at 4 °C to get the supernatants, which contained the nuclear fractions, and all samples were stored in liquid nitrogen until use. The protein concentrations were determined by enhanced BCA Protein Assay Kit with bovine serum albumin (BSA) as standard.

### Western blotting analysis

Protein samples were heated at 100 °C for 5 min with loading buffer containing 0.125 M Tris-HCl (PH 6.8), 20% glycerol, 4% SDS, 10% mercaptoethanol, and 0.002% bromphenol blue, then separated by sodium dodecyl sulfate-polyacrylamide gel electrophoresis (SDS-PAGE) using 10% acrylamide gels (50 μg per lane). Then, the proteins on the gel were transferred into a PVDF membrane using a wet transfer system, followed by blocking for 1 h in 3% BSA and then incubated overnight at 4 °C with the following primary antibodies: NLRP3 (1:500), ASC (1:200), GPER (1:500), Iba1 (1:1000), CD11b (1:1000), p65 (1:1000), bcl2 (1:200), cleaved-IL1β (1:200), cleaved caspase 1 (1:200), cleaved caspase 3 (1:1000), IL1RA (1:500), GAPDH (1:1000), CREB (1:1000), p-CREB (1:1000), and tubulin (1:500). The membranes were washed using 0.2% tween-20 in Tris-buffered saline (TBST) for at least 30 min at room temperature followed by incubation in HRP-conjugated secondary antibodies for 1 h at room temperature. Bound proteins were visualized using a CCD digital imaging system, and semi-quantitative analyses of the bands were performed with the ImageJ 1.49 analysis software. Band densities for the targeted proteins were normalized to loading controls (GADPH or β-tubulin). Normalized means were then expressed as fold changes of the corresponding value for control (sham operated) animals. A means ± SE was calculated from the data for graphical presentation and statistical comparison.

### Primary neuron culture

Primary hippocampal neurons were micro surgically isolated from SD rats at embryonic day 18 (E18) as our previous description [25]. Briefly, dissociated cells were plated on glass coverslips in presence of 10 μg/mL poly-d-lysine (Sigma)-coated 24-well culture plats at a density of 4.50 × 10^5^ cells/mL. Neurobasal medium supplemented with 2% B-27 (GIBCO, ThermoFisher Scientific, Milan Italy), 0.5 mM glutamine, 25 μM glutamate, penicillin/streptomycin (100 units/100 μg for mL), and an antimycotic agent, Amphotericin B was used for the maintenance of cell cultures.

On day 2 post-plating, half-volume medium was routinely replaced every 3 days, and neuron culture was maintained in a humidified incubator in an atmosphere of 5% CO_2_ at 37 °C. After 8 days, cells were submitted to the treatments. For G1-treatment, the neurons were incubated with the culture medium including 1 μM G1 final concentration for 30 min, 3 h, 6 h, 24 h, and 48 h, respectively. The time-point of 6 h was selected for pretreatment with G36 (10 μM) 20 min prior to stimulation with G1. Cottonseed oil with 0.1% DMSO final concentration was the control.

### Barnes maze test

Long-term spatial learning and memory was evaluated by the use of the Barnes maze, which is a widely accepted test of hippocampus-dependent spatial reference memory in rodents. The apparatus consisted of a circular platform of 120-cm diameter elevated 1 m above the floor, with 18 holes around the perimeter and a recessed chamber (black escape box also called target hole, 20 × 15 × 12 cm) located under one of the holes. During the testing, rats learn the spatial location of the target hole. The maze was surrounded by curtains on which there were visual cues to learn the position of the target hole. The maze testing was performed as described previously by our group with minor alterations [26, 27]. Briefly, the test included three parts, pre-training was carried out at 7 days of reperfusion, followed by 3 days (reperfusion 7 days, 8 days, 9 days) latency trial, and 24 h later, a probe trial was performed (reperfusion 10 days). During the maze testing, the room is lit with a bright flood incandescent light (500 W, 1000 lx) shining down on the maze center and a buzzer (85 dB) turning on. For the pre-training test, the rat was placed in the center of the open platform surface in a black colored cylindrical start chamber. After 10 s elapsed, the chamber was lifted and the rat was pre-trained to enter the escape box by guiding it to the escape box and remaining there for 2 min. Following the pre-training, the latency trial started and was repeated four times each day. At the beginning of each trial, the rat was placed in the same start chamber, and 10 s after the onset of the light and buzzer, the chamber was lifted and the rat was free to explore the maze. The trial ended when the rat entered the escape box, or after 3 min exploration, it failed to find the target hole. The light and buzzer were turned off once each trial ended, and the rat was allowed to stay in the chamber for 1 min for habituation. Trails were recorded by a camera located overhead of the platform, and the escape latency, escape velocity, and the time spend in the target quadrant (quadrant occupancy) were computerized using ANY-maze analyzer software. The platform was cleaned with 70% ethanol and dried with a blower fan after each trial. The probe trial was carried out on day 10 of reperfusion. In the trial, the escape box was removed and the time spent in the target quadrant where the escape box had been recorded during a 90-s period.

### Novel object recognition (NOR)

The apparatus for the NOR task consisted of an opaque box measuring 50 cm × 50 cm × 40 cm high. The test includes three stages: habituation training was carried out on day 11 and day 12 after ischemia, object familiarization trial was carried out on day 13 after ischemia, and then 24 h later, the NOR trial was conducted on day 14 after ischemia. The rats were first acclimated to the chamber for two consecutive days (5 min each day) prior to testing to explore the empty box. For the object familiarization testing, the rat was placed in the empty box for 1 min, and then, it was removed, and two identical objects (10 cm width, 10 cm height) were centrally fixed to the floor of the box situated 10 cm apart. The rat was then placed back in the box and allowed to explore for 5 min. The rat was repeatedly exposed to the same two identical objects twice a day at 60 min interval. Twenty-four hours later, the rat was returned to the object recognition box containing a copy of the object from the familiarization stage and a novel object that varied in color and size to test a long-term recognition memory. Object exploration was scored when the rat’s nose was within 2 cm of the object. Object exploration was not scored when the rat used the object to rear upward with the nose of the rat facing the ceiling. The time spent exploring each object and the discrimination index percentage (the percentage time spent exploring the new object) was recorded and analyzed using ANY-maze video tracking software as previously mentioned.

### Statistical analysis

Statistical analysis was performed using one-way analysis of variance (ANOVA) or two-way ANOVA followed by Student-Newman-Keuls tests to determine group differences. When only two groups were compared, a Student’s *T* test was used. Statistical significance was accepted at the 95% confidence level (*P* < 0.05). Data were expressed as means ± SE.

## Results

### GPER activation decreases GCI-induced neuroinflammation in the hippocampal CA1 region

**Fig. 1:**
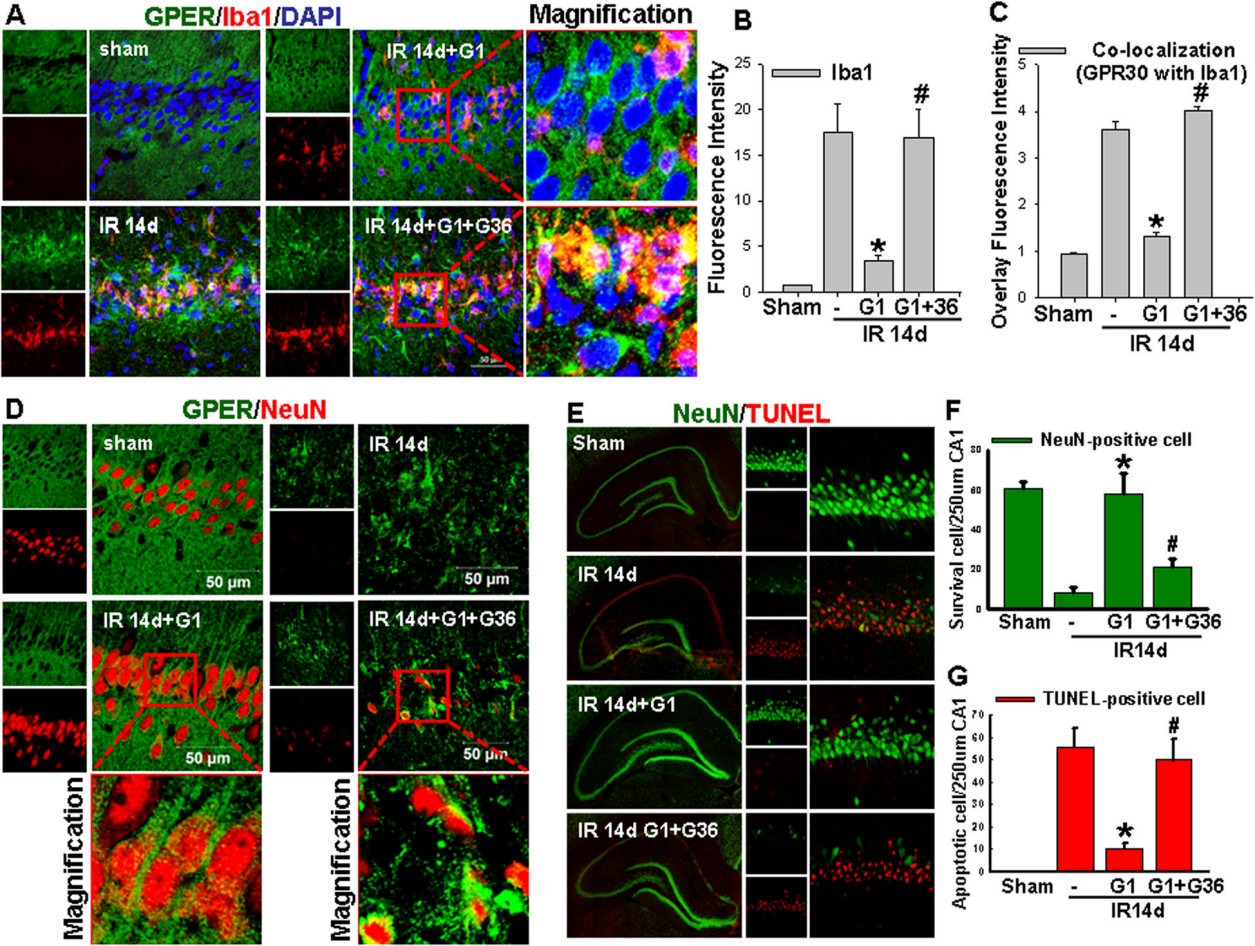
GPER activation attenuates neuroinflammation in the hippocampal CA1 region following GCI and enhances neuronal survival. **a** Representative images of double-immunofluorescent staining for GPER (green) and Iba1 (red) in indicated groups (yellow), indicating co-localization of GPER with Iba1, and DAPI (blue) counter stains nuclei. Quantitative analysis of Iba1 intensity (**b**) and co-localization of GPER with Iba1 intensity (ratio to sham) of hippocampal CA1 region (**c**). **d** Representative images of double-immunofluorescent staining for NeuN (red) and GPER (green) in indicated groups, which shows that G1 administration enhanced GPER immunoreactive levels in hippocampal neurons. **e** Representative photographs of NeuN staining (green) and TUNEL (red) in the indicated groups (reperfusion at 14 days). Quantification was performed by counting the number of NeuN-positive neurons (**f**) or TUNEL-positive (**g**) per 250 μm length in the medial CA1 pyramidal cell layer. Magnifications are zooms of the boxed areas. Scale bar, 50 μm. *n* = 7–8, ^*^P < 0.05 vs. IR group, ^#^*P* < 0.05 vs. G1-treated group. IR: ischemia reperfusion

In order to determine whether GPER signaling can exert anti-inflammatory effects after GCI, we first examined Iba1 expression and GPER distribution, as well as the effects of GPER agonist G1 or antagonist G36 at reperfusion 14 days after GCI in the hippocampal CA1 region. Iba1 was examined as it is a well-known marker of microglia that is upregulated upon activation of microglia due to inflammation. As shown in Fig. 1a–c, double-immunofluorescence staining for Iba1 (red) and GPER (green) in the hippocampal CA1 region revealed that ischemia/reperfusion (IR) 14 days induced a robust enhancement of Iba1 immunofluorescence intensity as well as higher co-localization (yellow) of Iba1 with GPER than that in non-ischemic (sham) animals. G1 administration markedly attenuated the Iba1 immunofluorescence intensity and the co-localization of Iba1 with GPER, while G36, a GPER antagonist, reversed the effect of G1 on Iba1 immunofluorescence intensity and co-localization of Iba1 with GPER in the hippocampal CA1 region after 14 days of reperfusion. DAPI (blue) staining was used to visualize the nucleus of cells. Furthermore, double-immunofluorescent staining of GPER (green) with NeuN (a marker of survival neurons, red) indicated that G1-treatment enhanced GPER immunoreactive levels in neurons in the hippocampal CA1 region (Fig. 1d). We next examined the effect of GPER activation upon apoptotic cell death in the hippocampal CA1 region at 14 days of reperfusion after GCI. G1 effect upon apoptosis in the hippocampal CA1 region at IR of 14 days was investigated using NeuN staining (green) and TUNEL analysis (red), and quantification of the results is provided in Fig. 1e–g. As expected, the number of survival neurons (NeuN-positive cells) was significantly decreased by G1 treatment, while the number of apoptotic cells (TUNEL-positive cells) was markedly increased compared to IR group animals, and G36 administration significantly abolished the anti-apoptotic effects of G1. Altogether, the results indicate that GPER exerts an anti-inflammatory regulatory effect that may contribute to its neuroprotective effect after GCI.

### NLRP3-ASC inflammasome activation in the hippocampal CA1 region after GCI is decreased by GPER activation

**Fig. 2:**
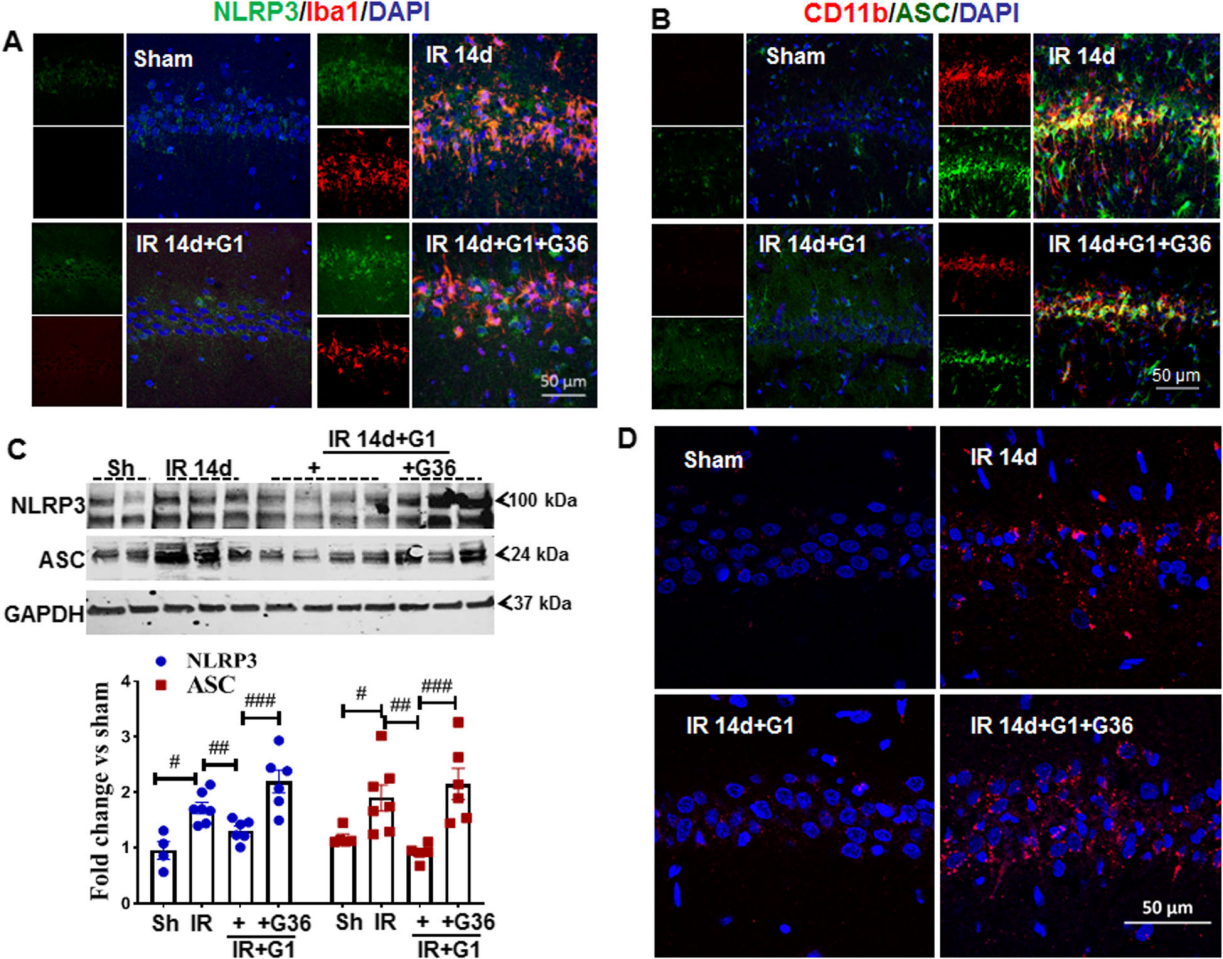
The GPER agonist, G1 attenuates protein expression of the NLRP3 inflammasome components in the hippocampal CA1 region following GCI. **a** Immunofluorescent staining of NLRP3 (green) and Iba1 (red), **b** CD11b (red), and ASC (green), showing the increased intensity of NLRP3 and ASC in the 14-day IR group, as compared to the G1-treated group, and G36 reversed the effect. Co-expression of NLRP3 with Iba1 or ASC with CD11b is indicated as yellow in the merged photomicrographs. **c** Western blot analysis of NLRP3 and ASC. Quantification was carried out according to the density of blot bands ratio to GAPDH. Data was expressed as means ± SE. GAPDH was used as a loading control. **d** Duolink analysis showed the interaction (red particles) of NLRP3 with ASC in both the 14-day IR and G36-treated groups compared to the sham or G1 group, DAPI (blue) counterstaining nucleus. ^#^*P* < 0.05 vs. sham group, ^##^*P* < 0.05 vs. 14-day IR group, ^###^*P* < 0.05 vs. G1-treated group. *n* = 4–5, scale bar 50 μm

The NLRP3 inflammasome is a major mediator of inflammation that is known to be activated by oxidative stress and inflammatory damage signals, resulting in caspase-1-dependent secretion of the pro-inflammatory cytokine IL-1β [28, 29]. We hypothesized that GPER signaling may regulate NLRP3 inflammasome activation as one of its anti-inflammatory mechanisms in the brain. To explore our hypothesis, we examined protein expression of the inflammasome components, NLRP3, and ASC in the hippocampal CA1 region at 14 days of reperfusion. Representative photomicrographs for double-immunofluorescent staining for NLRP3 (green) and Iba1 (red) are shown in Fig. 2a. The results show that the immunofluorescent intensity of NLRP3 and Iba1 are robustly enhanced and strongly co-localized in the 14-day IR group, as compared to the sham control animals. G1 treatment significantly attenuated the GCI enhancement and co-localization of NLRP3 and Iba1 levels, whereas pretreatment with G36 significantly reversed the G1 effects. Likewise, in Fig. 2b, double-immunofluorescent staining of ASC with CD11b, another marker of microglia, showed a similar pattern, with a robust elevation in protein levels of ASC and CD11b, as well as a very strong co-localization of the two proteins at reperfusion of 14 days, as compared to the sham group animals. G1 treatment significantly attenuated the GCI enhancement and co-localization of ASC and CD11b, whereas pretreatment with G36 significantly reversed the G1 effects. Furthermore, Western blot analysis results for NLPR3 and ASC (Fig. 2c) closely mirrored the findings from immunofluorescent staining of NLRP3 and ASC, as G1 prevented the elevation of NLRP3 and ASC on IR of 14 days, and G36 reversed this effect. It is well established that the recruitment and binding of ASC by NLRP3 is an absolute prerequisite for the activation of the NLRP3 inflammasome. Therefore, we performed an in vivo Duolink II in situ proximity ligation assay using primary antibodies for NLRP3 and ASC to detect the interaction between the two proteins. In Fig. 2d, Duolink puncta (red) in the representative photomicrographs indicates the interaction of NLRP3 with ASC in the hippocampal CA1 region. The results showed that G1 treatment significantly decreased Duolink puncta, as compared with the 14-day IR groups. However, pretreatment with G36 caused Duolink puncta to be increased again, reversing the effect induced by G1 administration. DAPI staining (blue) was also used to visualize the nucleus of cells. As a whole, the findings suggest that GPER decreases protein expression of NLRP3 and ASC and prevents the oligomerization of ASC and NLRP3 necessary for activation of the NLRP3 inflammasome after GCI.

### GPER activation inhibits “active” caspase 1 and IL1β expression in the hippocampal CA1 region after GCI

**Fig. 3:**
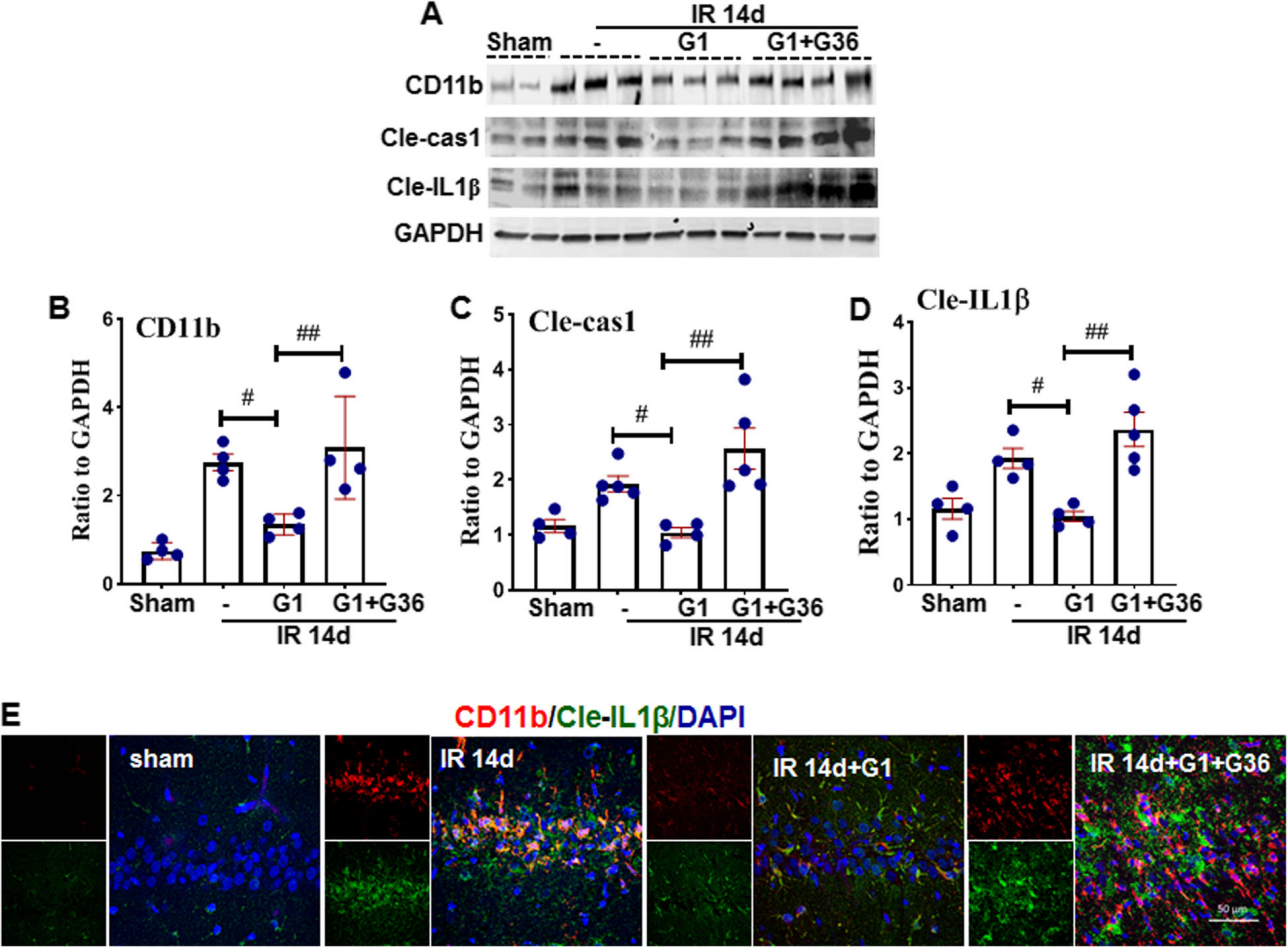
The effects of the GPER agonist, G1 upon caspase 1 activation and IL1β production in the hippocampal CA1 region following GCI. **a**–**d** Western blot analysis for CD11b, a marker of activated microglia, Cle-cas1 (cleaved caspase 1) and cleaved IL1β. Semi-quantitative analysis was carried out according to the band density of target protein ratio to that of loading control (GAPDH). Data was expressed as mean ± SE. ^#^*P* < 0.05 vs. 14-day IR group, ^##^*P* < 0.05 vs. G1-treated group, **e** Double-immunofluorescent staining for CD11b (red) and cleaved IL1b (green), showing a strong increase in CD11b and cleaved IL1b intensity in the 14-day IR animals compared to the G1-treated group, while G36 reversed the increase. Scale bar 50 μm, magnification 40× *n* = 4–5

It has been demonstrated previously that the NLRP3-ASC complex binds caspase 1, thus forming an active inflammasome complex (NLRP3, ASC, and cleaved caspase-1) that produces IL1β. The IL1β precursor is cleaved by “active” caspase 1 (Cle-caspase 1) to form mature IL1β (cleaved IL1β, Cle-IL1β) [28, 30, 31]. Thus, next, we investigated whether GPER activation could suppress activation of the NLRP3 inflammasome complex after GCI. As shown in Fig. 3a–d, Western blot analysis revealed that G1 markedly suppressed protein levels of activated caspase 1 (cle-caspase 1), cle-IL1β, and CD11b, as compared with the 14-day IR group, while G36 reversed the effects in the hippocampal CA1 region. Furthermore, double-immunofluorescent staining for cle-IL1β (green) and CD11b (red) confirmed the above Western blot analysis results and showed that cle-IL1β was strongly co-localization with CD11b in the 14-day IR and G1 + G36 groups, as compared to the sham or G1 groups in the hippocampal CA1 region (Fig. 3e). These findings suggest that NLRP3 inflammasome activation occurs predominately in activated microglia and is suppressed by GPER activation.

### GPER regulates expression of endogenous IL1β receptor antagonist (IL1RA) in the hippocampal CA1 region after GCI

**Fig. 4:**
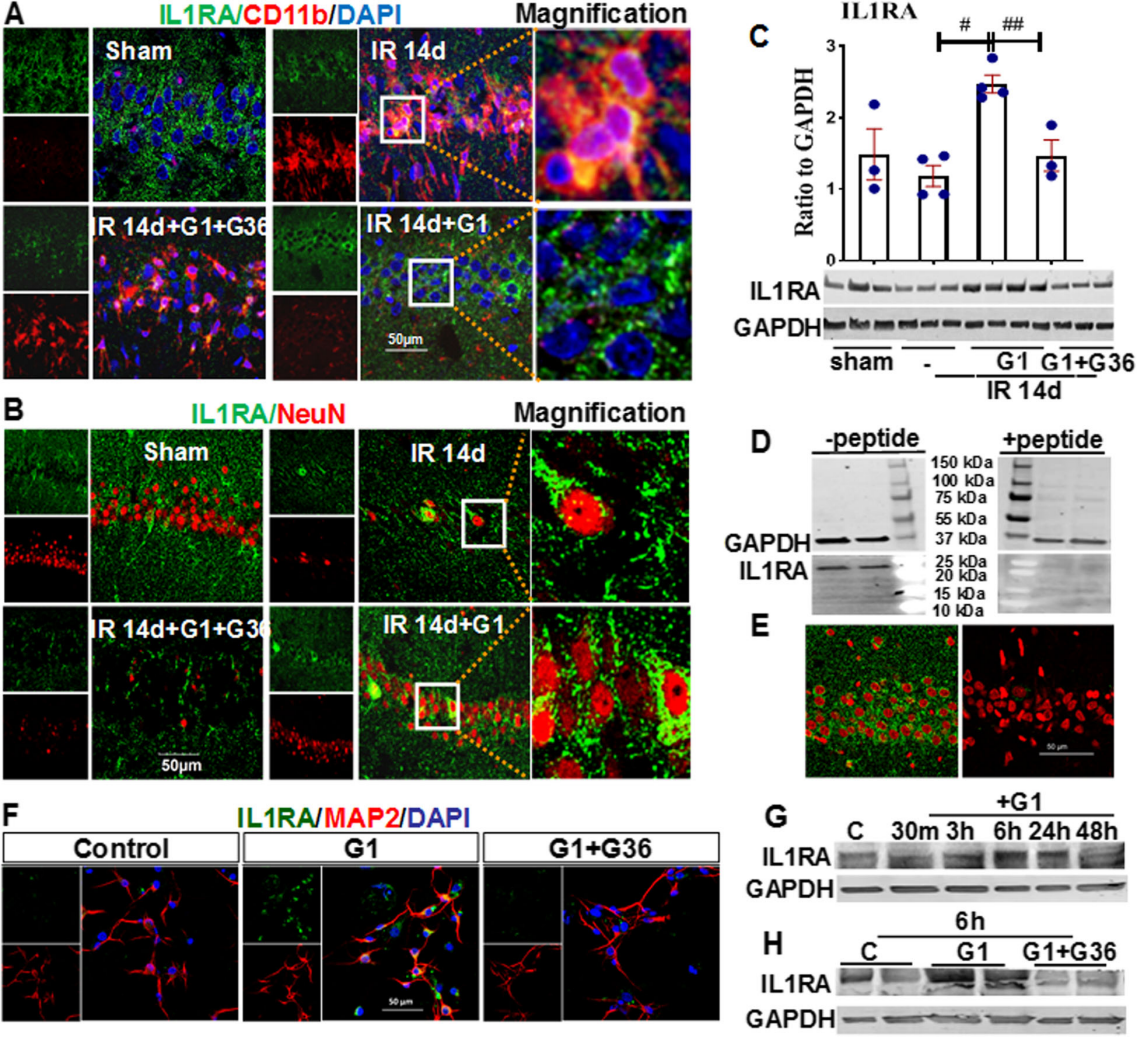
The effects of the GPER agonist, G1 upon IL1RA immunoreactive protein levels in the hippocampal CA1 region following GCI. **a** Representative images of double-immunofluorescent staining for **a** IL1RA (green) and CD11b (red), **b** IL1RA (green) and NeuN (red). Co-localization of IL1RA immunoreactive protein with the microglia marker, CD11b, and neuronal survival marker, NeuN, is shown in magnification images. DAPI (blue) counter stain for the cell nucleus. **c** Western blot analysis for IL1RA. ^#^*P* < 0.05 vs. 14-day IR group, ^##^*P* < 0.05 vs. G1-treated group. *n* = 4 in sham and G1 groups and *n* = 5 in IR and G36 + G1 groups. **d**, **e** The specificity of IL1RA antibody was addressed using Western blot and immunofluorescent staining of IL1RA, and DAPI (red) counter stain for cell nucleus. **f**–**h** Effect of G1 on IL1RA protein expression in primary hippocampal neurons. Representative photographs of double-immunofluorescence staining for IL1RA (green) and MAP 2 (red) at 6 h after G1 or G1 + G36-administration, DAPI (blue) counter stain for cell nucleus (**f**). Western blot analysis showed the IL1RA protein expression in indicated groups (**g**, **h**). × 40 magnification, scale 50 μm

In addition to suppressing NLRP3 inflammasome activation, we hypothesized that GPER activation might also exert anti-inflammatory effects by regulating expression of IL1RA, an endogenous IL1β receptor antagonist. We thus examined IL1RA protein expression in the hippocampal CA1 region using immunofluorescent staining and Western blot analysis. Representative photomicrographs of double-immunofluorescent staining results for IL1RA (green) and CD11b (red), or IL1RA (green) and NeuN (red) in the hippocampal CA1 region, are shown in Fig. 4a, b. The results reveal that IL1RA immunoreactive levels are strongly decreased in the 14-day IR group as compared to the sham controls. Interestingly, G1 strongly upregulated IL1RA immunoreactive protein levels, as compared to the 14-day IR group, and G36 treatment prevented this effect of G1, while in the 14-day IR group, the IL1RA immunoreactive protein present was found to be co-localized with CD11b in microglia. In contrast, using NeuN as a neuronal marker, we found that G1 strongly upregulated IL1RA immunoreactive protein levels in hippocampal CA1 neurons, and this effect was blocked by G36 (Fig. 4a, b). Western blot results for IL1RA showed that G1 treatment significantly elevated IL1RA protein levels compared with the 14-day IR group, and G36 reversed this effect of G1 (Fig. 4c). The specificity of the IL1RA antibody used in the study was confirmed by pre-incubation with or without blocking peptide of IL1RA. Western blot for IL1RA using samples from hippocampal CA1 region of sham animals are shown in Fig. 4d. The results demonstrated that in the absence of blocking peptide pretreatment, there was a clear band for IL1RA at around 25 KDa (left line). In contrast, pretreatment with blocking peptide completely blocked the 25 KDa band of IL1RA. In addition, the blocking peptide perfectly abolished staining for IL1RA in the hippocampal CA1 region (green, Fig. 4e), and DAPI (red) staining was used to visualize the nucleus of cells. The results demonstrate the specificity of the IL1RA antibody. They also demonstrate that G1 strongly induces IL1RA immunoreactive protein levels after GCI in neurons in the hippocampal CA1 region, thus potentially “armoring” the neurons against IL1β-induced inflammatory damage and cell death. In order to detect whether G1 directly induces IL1RA protein expression, we performed primary hippocampal neuron cultures and treated the neurons with GPER agonist G1. Pretreatment with G1 was for 6 h, and G36 (10 μM) was administered 20 min prior to stimulation with G1. Cottonseed oil with 0.1% DMSO final concentration was as the control (C). Western blot analysis showed that there was no significant change in IL1RA protein expression at 30 min and 3 h after G1-treatment, whereas G1 markedly enhanced IL1RA expression at 6 h and 24 h, as compared to the control group (Fig. 4g). G36-administratment reversed the effect induced by G1 at the 6 h time-point (Fig. 4h). Furthermore, immunofluorescent staining for MAP 2 (neuron marker, red) and IL1RA (green) further confirmed the above results (Fig. 4f).

### IL1RA knockdown abolishes the anti-inflammasome effect induced by GPER activation in the hippocampal CA1 region following GCI

**Fig. 5:**
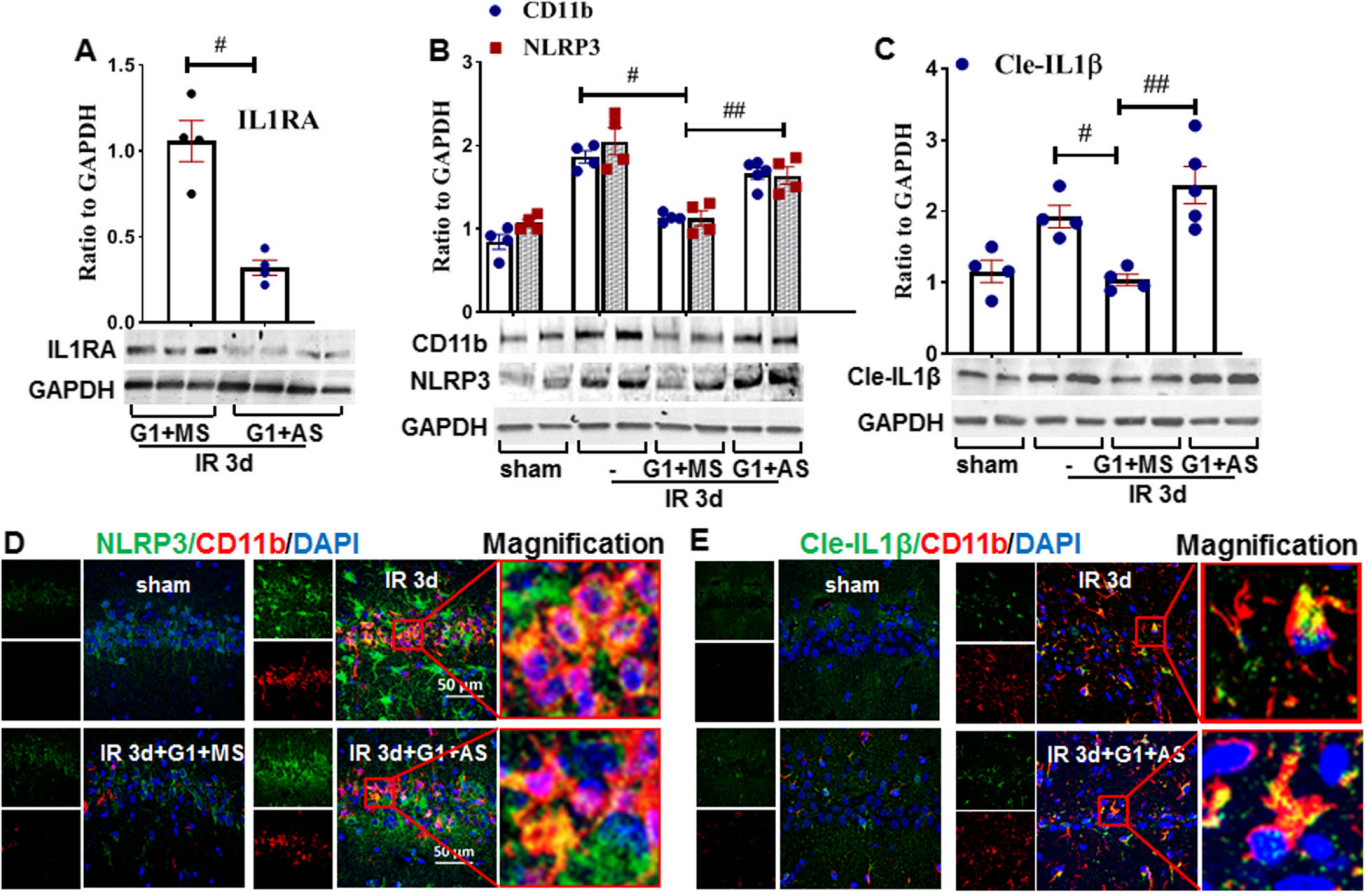
IL1RA knockdown abolishes the anti-inflammatory effects of GPER activation at 3 days of reperfusion in the hippocampal CA1 region following GCI. **a** Western blot analysis showed that IL1RA antisense oligodeoxynucleotide (AS) knockdown significantly decreased IL1RA protein expression (*n* = 4 in each group). Samples for Western blot were from sham, IR, G1 plus IL1RA missense oligodeoxynucleotide (MS), and G1 plus AS. Antibodies of CD11b (*n* = 4 in sham, IR, G1 groups, and *n* = 5 in G36 + G1-treated group), NLRP3 (**b**) (*n* = 4 in each group), cleaved-IL1β (**c**) (*n* = 4 in sham, IR, G1 groups, and *n* = 5 in G36 + G1-treated group), and loading control GAPDH. ^#^*P* < 0.05 vs. 3-day IR group, ^##^*P* < 0.05 vs. G1 + MS group. Double staining for NLRP3 (green) and CD11b (red) (**d**) or cleaved-IL1b (green) and CD11b (red) (**e**) in the indicated groups. × 40 magnification, scale bar 50 μm

We next examined the role of IL1RA in GPER regulation of the NLRP3 inflammasome after GCI. To accomplish this, we centrally administered IL1RA antisense oligonucleotides (IL1RA-AS) into the lateral ventricle to knockdown IL1RA in the hippocampal CA1 region. First, we investigated the effectiveness of IL1RA-AS using G1-treated samples. Western blot analysis results for IL1RA are shown in Fig. 5a, which revealed that IL1RA protein expression was markedly decreased by IL1RA-AS, as compared to the G1 + MS control group in the hippocampal CA1 region, thus validating the knockdown effectiveness of the IL1RA-AS. We next determined the effect of IL1RA-AS on the ability of G1 to regulate protein expression of CD11b, NLRP3, and Cle-IL1β. As shown in Fig. 5b, c, Western blot analysis indicated that G1-treatment (G1 + MS) significantly decreased both NLRP3 and cle-IL1β protein expression in the hippocampal CA1 region, as compared with the 3-day IR group, and this effect was profoundly reversed by IL1RA-AS treatment. Furthermore, double-immunofluorescence staining for NLRP3 (or cle-IL1β) with CD11b showed that similar to 3-day IR group animals, IL1RA-AS administration animals had a markedly enhanced number of CD11b + microglia (red) with fewer and much thicker increase in the size of the cell bodies in the CA1 region, while G1 + MS control animals displayed very few CD11b + microglia with a small cell body and elaborated thin processes (Fig. 5d, e). As expected, immunoexpression of NLRP3 was in accordance with the change of CD11b, and co-localization of NLRP3 (green) with CD11b (red) (Fig. 5d) or cle-IL1β (green) with CD11b (red) (Fig. 5e) was seen in the 3-day IR and IL1RA-AS treatment groups rather than in the G1 + MS control and sham groups. As a whole, the results suggest that IL1RA may be an important factor in mediating anti-inflammasome activities of GPER following GCI in the hippocampal CA1 region.

### G1 inhibits NF-κB-P65 nuclear recruitment in an IL1RA-dependent manner

**Fig. 6:**
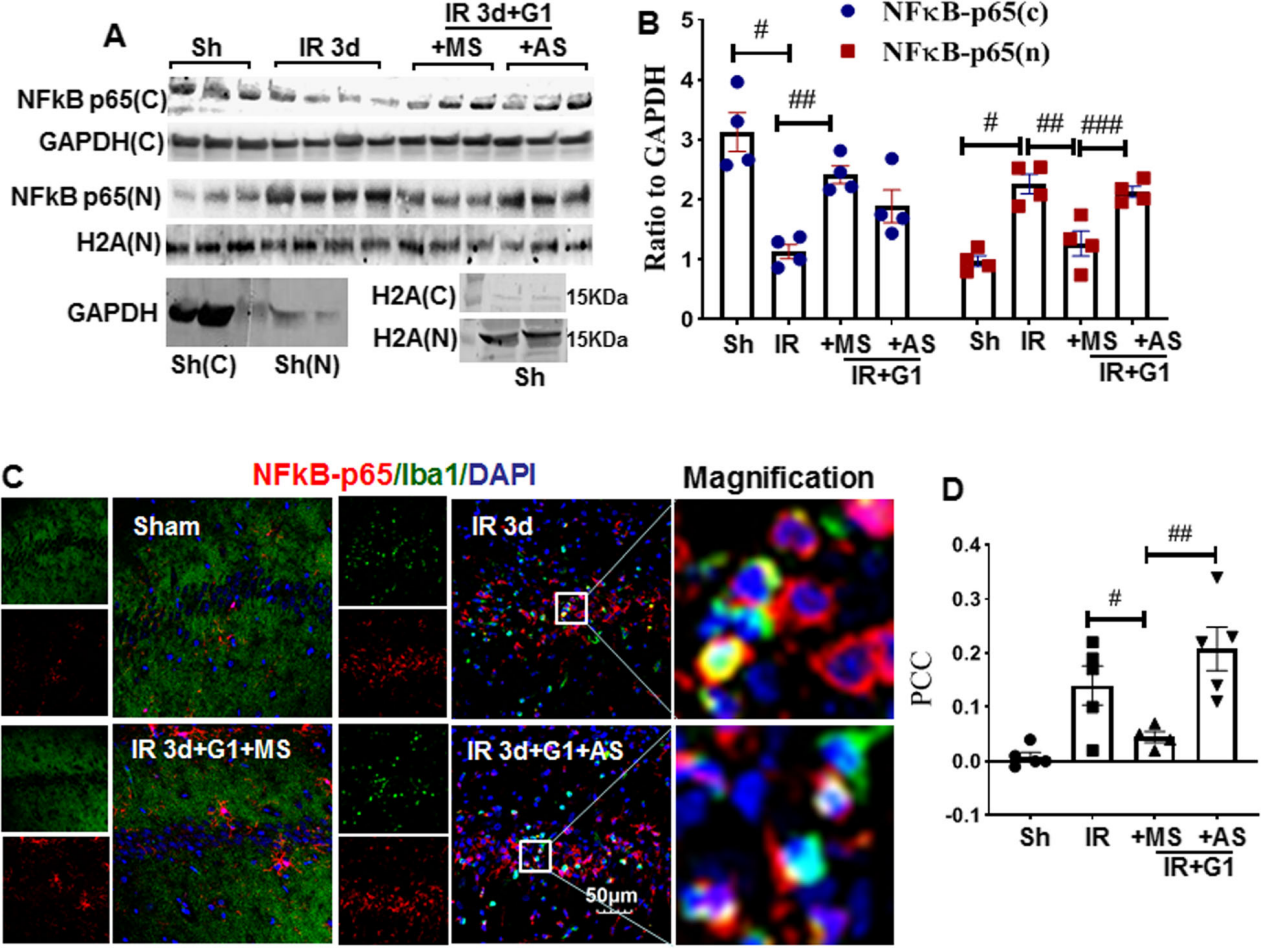
IL1RA knockdown reverses G1 regulation of NF-κB signaling in the hippocampal CA1 region following GCI. **a**-**b** Western blot analysis for NF-κB p65 in cytoplasm and nucleus fractions, and GAPDH, H2A as the loading controls of cytoplasm and fraction, respectively. ^#^*P* < 0.05 vs. sham group, ^##^*P* < 0.05 vs. 3-day IR group, and ^###^*P* < 0.05 vs. G1 + MS group. **c** Double straining of NF-κB-p65 (red) and Iba1 (green). **d** Colocalization analysis of NFkB-P65 and Iba1 by Pearson Correlation Coefficient (PCC). ^#^*P *< 0.05 vs. 3-day IR group, and ^##^*P *< 0.05 vs. G1 + MS group. × 40 magnification, scale bar 50 μm, *n* = 4–5

NF-κB is a major transcription factor that has been implicated as a critical regulator of gene expression in inflammation, particularly in IL-1 production and secretion [32]. Nuclear migration of P65, a subunit of NF-κB, is pivotal for its activity, and there is evidence that inhibition of NF-κB inhibits de novo pro-IL1β [33, 34]. We thus examined the effects of G1 on p65 nucleus translocation at IR of 3 days and further elucidated whether IL1RA is a critical mediator of the process. As shown in Fig. 6a, b, Western blot analysis indicated that p65 protein levels were significantly decreased in the cytoplasm fraction at the 3-day IR group compared to the sham group, and G1 treatment prevented the decrease. An opposite pattern was observed in the nuclear fraction with p65 protein levels showing a robust enhancement in the 3-day IR group, as compared to the G1 + MS control group. Intriguingly, IL1RA-AS profoundly increased p65 protein levels compared to G1 + MS control, although there was no statistical change in the cytoplasm fraction between the two groups. Additionally, Western blot of GAPDH and H2A from both the cytoplasmic and nuclear compartments of sham samples demonstrated the purity of both subcellular fractions (Fig. 6a). Double-immunofluorescent staining for p65 (red) and Iba1 (green) (Fig. 6c, d) showed a robust enhancement of p65 in Iba1+ microglia cells and obviously nuclear expression in the 3-day IR and IL1RA-AS groups, as compared to the sham and G1 + MS groups. As a whole, the results indicate that G1 regulates NF-κB /p65 protein nuclear translocation in an IL1RA-dependent manner.

### Central knockdown of IL1RA protein abolishes the G1-induced anti-apoptotic effect in the hippocampal CA1 region after GCI

**Fig. 7:**
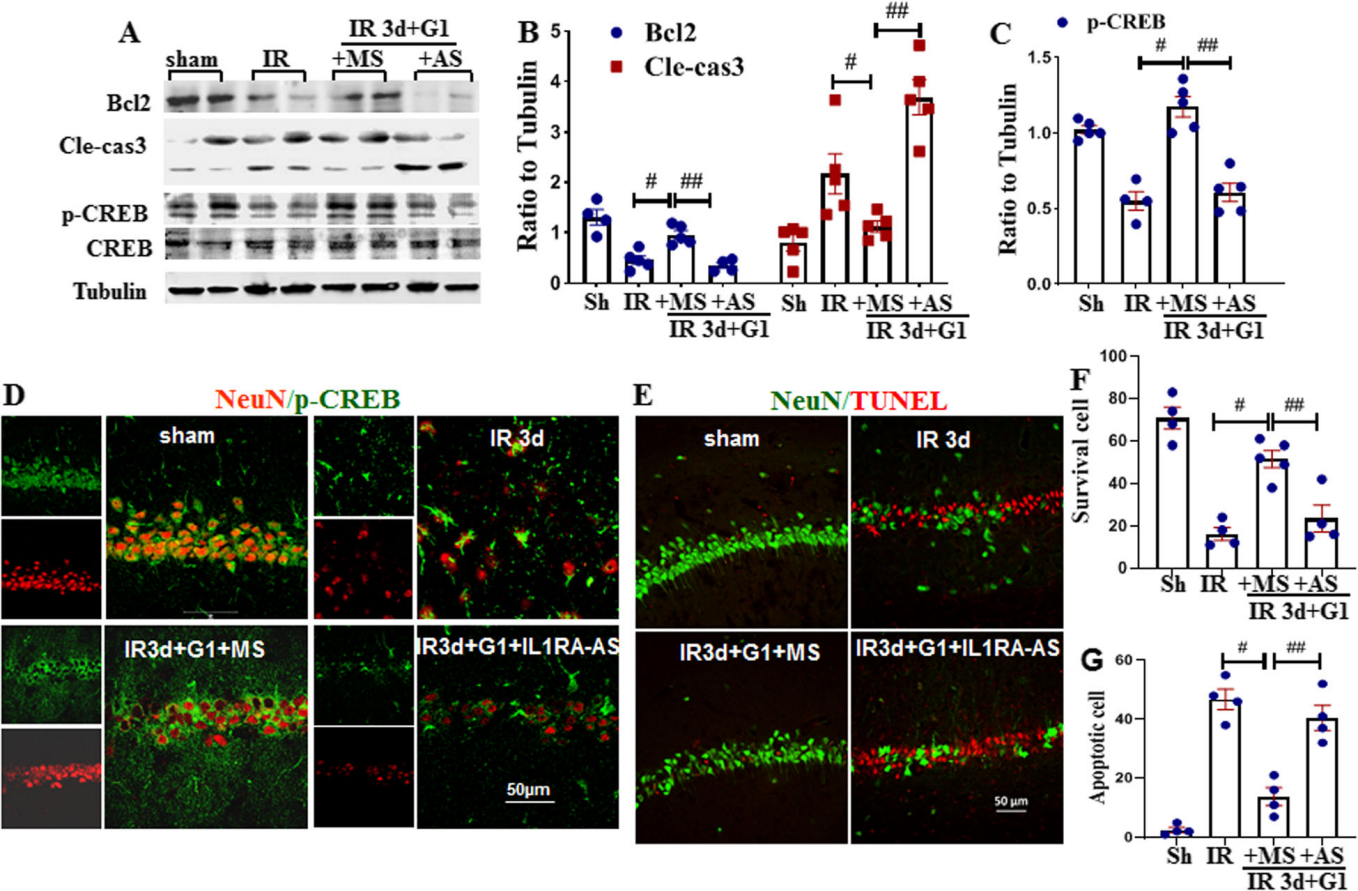
IL1RA knockdown abolishes the neuroprotective effect of G1 following GCI in the hippocampal CA1 region. **a** Western blot analysis of anti-apoptotic protein Bcl2, CREB activation, and an executioner of apoptosis, cle-cas3 (cleaved caspase 3). **b** Semi-quantitative analysis was carried out according to the band density of Bcl2 or cle-cas3 and p-CREB (**c**) ratio to that of loading control (tubulin). **d** Representative images of double-immunofluoscence staining for NeuN (red) and p-CREB (green). Data was expressed as mean ± SE. ^#^*P* < 0.05 vs. 3-day IR group, ^##^*P* < 0.05 vs. G1 + MS-treated group. *n* = 4–5. **e**–**g** Representative photomicrographs of NeuN staining (green) and TUNEL analysis (red) in the indicated groups. *n* = 5, scale bar 50 μm

Neuroinflammation is known to occur in parallel with mitochondrial dysfunction and apoptosis. Therefore, we next sought to determine whether G1 regulated mitochondrial apoptosis signaling in the current study, and whether this was dependent upon IL1RA. Western blot analysis showed that Bcl2, an anti-apoptotic factor was significantly enhanced by G1, as compared to IR of 3 days, and IL1RA-AS knockdown attenuated this effect. Cleaved caspase 3, which is known as an apoptotic executor, displayed an opposite pattern as that of Bcl2 (Fig. 7) a, b. The transcription factor, CREB, is known to regulate IL1RA expression in the brain [35]. Examination of CREB phosphorylation by Western blot analysis showed that G1-treatment significantly enhanced CREB phosphorylation (activation) compared to the 3-day IR group, while ILIRA knockdown markedly abolished the effect (Fig. 7c). Double-immunofluorescence staining for NeuN and p-CREB mirrored the Western blot results, revealing that G1 enhanced p-CREB in hippocampal CA1 region neurons and that IL1RA-AS reversed this effect (Fig. 7d). Next, apoptosis detection was carried out using TUNEL analysis, and at the same time, NeuN immunofluorescence staining was performed as a measure of neuronal survival in the hippocampal CA1 region. Figure 7e shows the representative photographs of NeuN (green) and TUNEL (red) staining in indicated groups, while semi-quantitative analysis of the staining is shown in Fig. 7f, g. The results revealed that G1 + MS prevented neuron apoptosis and increased the number of surviving neurons as compared to IR of 3 days, whereas IL1RA-AS significantly abolished these neuroprotective and anti-apoptotic effects of G1. Taken together, the findings suggest that GPER activation enhances the defense mechanism in hippocampal neurons after GCI and that this effect requires upregulation of IL1RA.

### Central knockdown of IL1RA reverses G1-induced cognitive enhancement following GCI

**Fig. 8:**
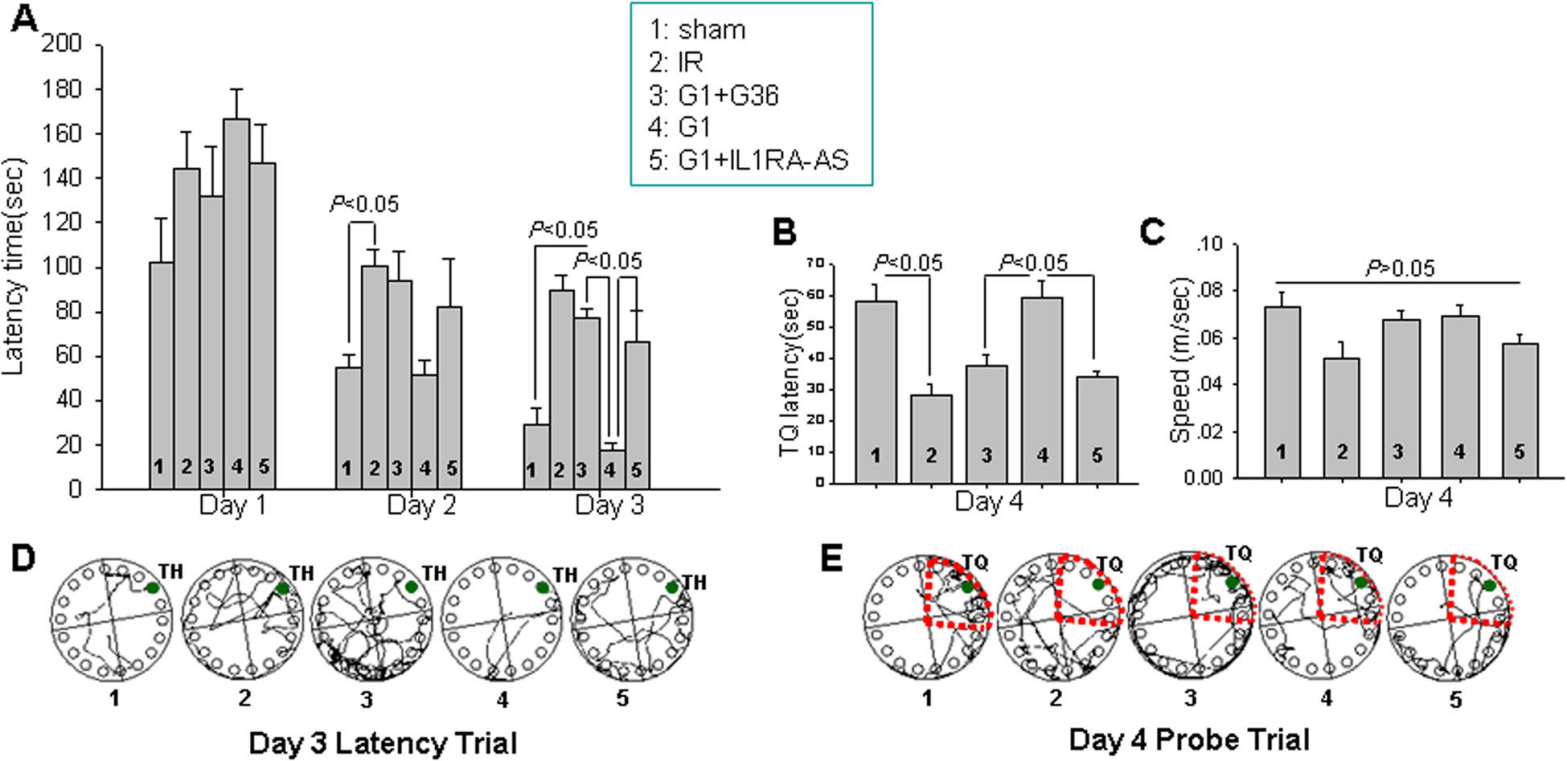
G1 enhancement of spatial learning and memory after GCI are abolished by G36 treatment or IL1RA knockdown. **a** Barnes maze was performed to examine spatial learning and memory. **a** The time (s) spent finding target hole (TH) at days 7, 8, and 9 after ischemia insult. **b** Exploration time spent in the target quadrant (TQ) that initially contained the TH at day 10 following reperfusion. **c** Moving speed of the rats in the probe trail on the fourth day of the test. **d**, **e** Representative traces indicating the sample paths of the rats from the maze latency trials and probe trials. Data are expressed as mean ± SE from 5 different animals. *P* < 0.05 considered as statistic difference between the groups. 1 sham, 2 IR, 3 G1 + G36, 4 G1, 5 G1 + IL1RA-AS

We next examined whether G1 treatment preserved hippocampal-dependent cognitive function following GCI and whether this effect was dependent upon upregulation of IL1RA. We used the Barnes maze to examine hippocampal-dependent cognitive function at 7–10 days after reperfusion as it is a widely used test to assess spatial reference learning and memory [36, 37]. As shown in Fig. 8a, there was no significant change on day 1 of the latency trial among the groups, while on day 2 and day 3, reperfusion at 9 days resulted in a significant increase in latency to find the target hole (Fig. 8a), as compared to the sham group, with a significant decrease in the time spent in the escape box quadrant (Fig. 8b). On day 2 of the latency trial, as compared to the IR group, G1 administration decreased the time to find the escape box but this was not statistically significant, while on day 3, G1 significantly decreased the time to find the target hole (Fig. 8a) and increased the time spent in exploring the quadrant where the escape box had been (Fig. 8b). Importantly, administration of either the GPER antagonist G36 or IL1RA-AS reversed the cognitive improvements induced by G1, as evidenced by G36- and IL1RA-AS-treated animals exhibiting a significantly enhanced latency to find the target hole and significantly decreased time spent in the quadrant where the escape box had been, as compared to only G1-treated animals (Fig. 8a, b). Further studies revealed no difference in the exploring speed of the animals (Fig. 8c). Representative tracings indicating sample paths of the rats from the latency trial and probe trial are shown in Fig. 8d, e.

**Fig. 9:**
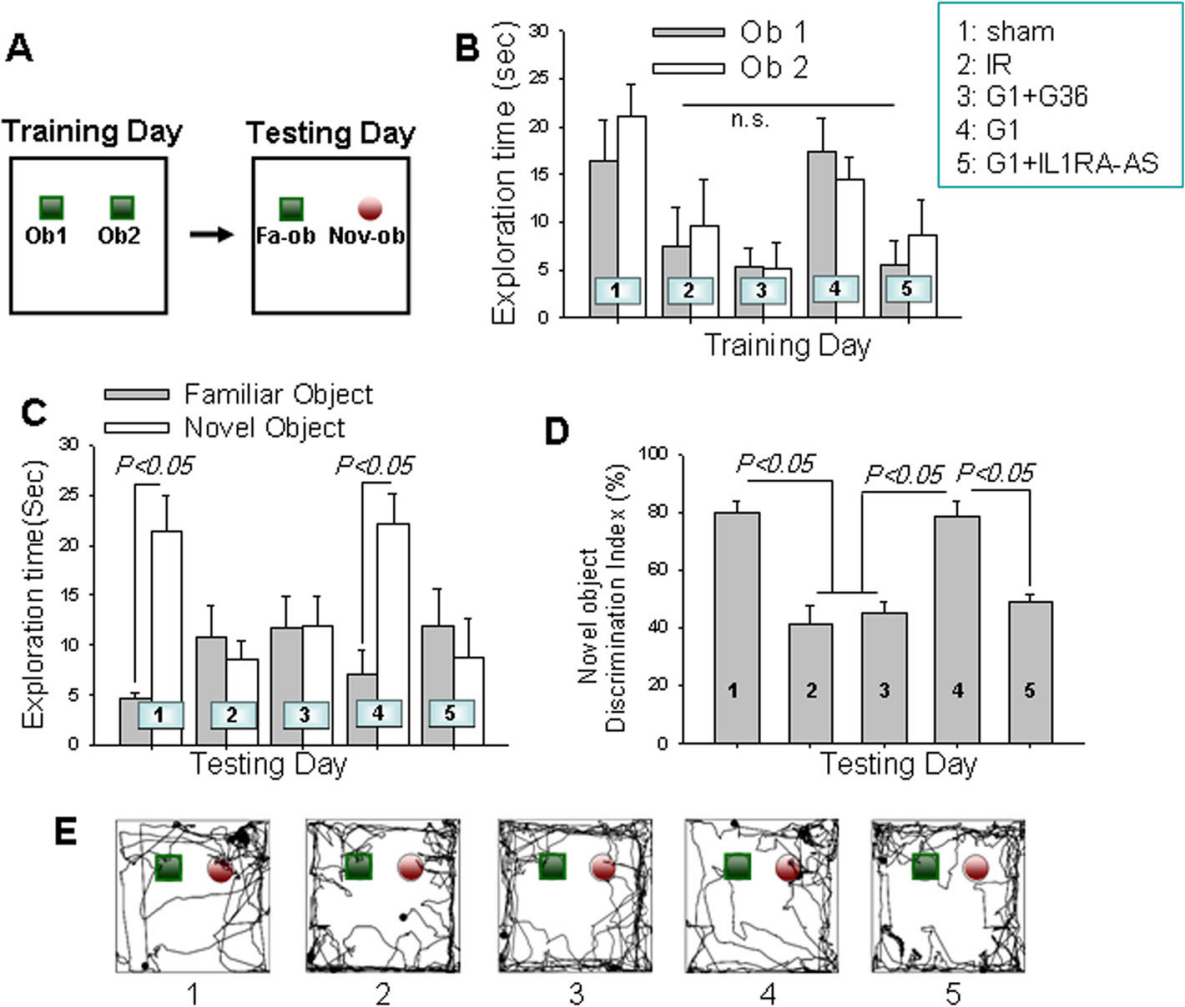
G1 enhancement of reference memory after GCI is abolished by G36 treatment or IL1RA knockdown. The novel object recognition (NOR) test was performed following GCI. Five-minute NOR tests at 13 and 14 days after 12-min ischemia were performed to monitor the long-term memory (**a**). **b** Time spent exploring the two familiar objectives on the training day. **c** The time spent exploring each object (familiar object and novel object) and **d** the discrimination index percentage (the time spent exploring novel object divided by total time spent). **e** Representative traces of the indicated groups on the testing day. All the data are expressed as means standard error from 6 to 7 animals in each group. ^*^P < 0.05 considered as a statistic difference between the groups. n.s., no significant change; Ob, object; Fa-ob, familiar object; Nov-ob, novel object

In order to further address the cognitive improvement effects of G1-induced IL1RA signaling, we next performed the novel object recognition test to assess hippocampus-dependent working memory in the rats [38]. Figure 9a shows the protocol of the training day (reperfusion at 13 days) presenting two identical objects in the box, and the testing day (reperfusion at 14 days) presenting one familiar object (Fa-ob) and a novel object (Nov-ob). Figure 9b demonstrates that the time spent by each group of animals in exploring the two objects had no significant differences on training day. However, on testing day, sham and G1-treated animals spent a significantly longer time exploring the novel object than the familiar object. In contrast, the time spent on the familiar and novel objects in IR, G1 + G36-treated and G1 + IL1RA-AS-treated animals showed no statistically significant differences (Fig. 9c). Notably, the increased discrimination index of G1 was profoundly decreased by G36 and IL1RA-AS, demonstrating that the effective role of G1 in improving long-term learning and recognition memory following GCI is mediated by GPER and IL1RA (Fig. 9d). Representative tracings indicating sample paths of the rats from testing day are shown in Fig. 9e.

## Discussion

The current study provides several important findings. First, it demonstrates that GPER activation exerts potent anti-inflammatory effects in the hippocampus after GCI to reduce microglia activation, suppress NLRP3 inflammasome activation, and downstream active IL-1β generation and signaling. Secondly, it demonstrates that GPER activation enhances defense mechanisms in neurons by profoundly upregulating the anti-inflammatory protein and IL1RA in neurons in the hippocampal CA1 region after GCI. Thirdly, it demonstrates that IL1RA is critical for mediating GPER’s anti-inflammatory, neuroprotective, anti-apoptotic, and cognitive-preserving effects after GCI.

The results of our study demonstrate a profound anti-inflammatory and neuroprotective effect of GPER activation in the hippocampal CA1 region of ovariectomized rats after GCI. Our data suggest that these effects of GPER activation are mediated through effects on both neurons and microglia. Indeed, GPER was shown in this study and other studies to be strongly expressed in both neurons and microglia in the hippocampus and other brain areas [14, 39–42]. To our knowledge, our study is the first to demonstrate that administration of the GPER agonist, G1 to ovariectomized rats, can markedly decrease protein expression and prevent the oligomerization of ASC and NLRP3 in microglia in the hippocampal CA1 region after GCI. These effects of G1 were reversed by the GPER antagonist G36, demonstrating the critical role of GPER for the anti-inflammatory actions of G1 in the hippocampus. The suppression of NLRP3 and ASC expression and oligomerization by G1 after GCI was correlated with significant inhibition of IL-1β, a major pro-inflammatory product of the NLRP3 inflammasome. Elevation of IL-1β in the brain has been shown to inhibit synaptic strength and long-term potentiation in vivo [43], and its administration in vitro is neurotoxic [43]. In addition, treatment with an IL-1β neutralizing antibody has been shown to enhance functional cognitive recovery after GCI [44]. In agreement with these results, NLRP3 knockout mice and NLRP3 inhibitor-treated mice have likewise been shown to have significantly reduced infarct size and improved neurological outcome after cerebral ischemia [45–47]. Based on these findings, we propose that GPER inhibition of NLRP3 inflammasome activation and IL-1β in microglia could contribute, in part, to the anti-apoptotic and cognitive-preserving effects of GPER after GCI.

In this study, we only examined the role of GPER in ovariectomized female animals. It is unclear if GPER activation would exert a similar anti-inflammatory role after GCI in male animals. A review of the scientific literature reveals there is some controversy on whether sex differences exist in GPER actions after cerebral ischemia. For instance, Broughton et al. reported that G1 was only protective in ovariectomized females and not males following focal cerebral ischemia in mice [48]. Moreover, focal cerebral ischemia has been shown to induce GPER in the male mouse brain without altering expression in females [49]. However, another group which used cardiac arrest to induce GCI found that G1 was strongly neuroprotective in male animals [50]. The reasons for the discrepant results are not clear, but it could be due to different ischemia models, different doses, routes of administrations, or pretreatment periods for G1 administration used in the studies. Further work will be needed to more fully clarify potential sex differences in GPER actions in the ischemic brain.

In addition to its anti-inflammatory effects upon microglia, our study identified an additional novel mechanism by which GPER activation could potentially “armor” healthy neurons from inflammatory insults after cerebral ischemia. Using the agonist G1, we showed that GPER activation can markedly elevate expression of IL1RA in hippocampal neurons after GCI. IL1RA has been identified as a natural physiologically occurring negative regulator of inflammation that protects cells from insult [51, 52]. Thus, G1 induction of IL1RA in hippocampal neurons could serve to protect hippocampal neurons against the damaging effects of IL-1β after GCI. In support of this possibility, our study found that knockdown of IL1RA via central administration of IL1RA antisense oligonucleotides significantly attenuated the neuroprotective, anti-apoptotic, and cognitive-enhancing effects of G1 after GCI. This finding suggests that the elevation of IL1RA by G1 is essential for GPER’s beneficial neuroprotective and cognitive effects after GCI.

Our finding of G1 enhancing IL1RA in neurons is intriguing as most studies have identified IL1RA as being a microglia-generated protein [53, 54], and few have ever studied its expression in neurons. However, treatment with a FDA-approved lipid-lowering drug, gemfibrozil, has been previously reported to directly upregulate IL1RA in mouse cortical neurons in vitro, and this effect strongly protected the neurons from IL-1β insult [35]. Furthermore, gemfibrozil induction of IL1RA in neurons was dependent upon enhanced PI3K-Akt signaling and CREB-regulated transcription of IL1RA [35]. We propose that GPER activation may directly regulate IL1RA in neurons via the same mechanism as we previously reported that G1 rapidly elevates PI3K-Akt signaling in hippocampal neurons after GCI [5], and the current study showed that GPER activation also enhanced CREB activation, as indicated by its increased phosphorylation. Consistent with this suggestion, previous studies have confirmed that GPER is expressed in extranuclear locations in hippocampal neurons, such as the plasma membrane, endoplasmic reticulum, and in dendrites [40, 55], and thus is well positioned to exert rapid extranuclear signaling. However, we cannot rule out the possibility that G1 could act indirectly in another cell type to induce IL1RA in neurons.

Finally, in addition to blocking the neuroprotective effect of G1, we found that IL1RA knockdown also attenuated the anti-inflammatory effect of G1 after GCI. Specifically, G1 ability to suppress NLRP3, cleaved IL-1β, and NF-κB activation in the hippocampal CA1 region after GCI was significantly attenuated by IL1RA knockdown. While the mechanism of this effect remains to be elucidated, we propose that these effects could be due to loss of G1-induced IL1RA “armoring” of neurons, leading to their increased damage and/or demise and release of signals that can activate the NLRP3 inflammasome. In support of this possibility, neuronal damage is known to induce release of danger-associated molecular patterns (DAMPs) such as ATP, ROS, DNA, and HMGB1 that can bind to Toll-like receptors (TLRs) or other receptors on microglia and induce NLRP3 inflammasome activation and active IL-1β production in microglia [56, 57]. Thus, the increased release of DAMPs by damaged neurons could lead to microglia activation and explain why the anti-inflammatory effects of G1 were reversed by IL1RA knockdown.

## Conclusion

The findings of this study shed a new light on the role and underlying mechanisms of GPER control of neuroinflammation after GCI and the potential contribution of these effects to the neuroprotective actions of GPER. As such, our study demonstrates for the first time that GPER’s anti-inflammasome, anti-apoptotic, and cognitive-preserving effects in the hippocampal CA1 region after GCI involve upregulation and mediation by the potent anti-inflammatory factor, IL1RA.

## Data Availability

All data generated or analyzed during this study are available from the corresponding author on reasonable request.

## Abbreviations

(GPER/GPR30): G-protein-coupled estrogen receptor
4-VO: Four-vessel occlusion
ASC: Apoptosis-associated speck-like protein
CAA: Common carotid arteries
CREB: cAMP-response element binding protein
E2: 17β-estradiol
GCI: Global cerebral ischemia
IL1RA: Interleukin-1β receptor antagonist
IL-1β: Interleukin-1β
NF-κB: Nuclear factor kappa-light-chain-enhancer of activated B cells
NF-κB: Nuclear factor kB
NLRP3: NACHT-, LRR- and PYD-containing protein 3
NOR: Novel object recognition
PLA: Proximity ligation assay
